# ASSOCIATIONS BETWEEN NEIGHBORHOOD QUALITY AND COGNITIVE FUNCTION ARE MODERATED BY INTERPERSONAL STRESSOR CONTROL

**DOI:** 10.1093/geroni/igae098.0511

**Published:** 2024-12-31

**Authors:** Eric Cerino, Amanda Goldtooth, Raechel Livingston, Chloe Horowitz, Dakota Witzel, Jonathan Rush, Megan McCoy, Michael McCarthy

**Affiliations:** Northern Arizona University, Flagstaff, Arizona, United States; Northern Arizona University, Flagstaff, Arizona, United States; Northern Arizona University, Flagstaff, Arizona, United States; Northern Arizona University, Flagstaff, Arizona, United States; South Dakota State University, Brookings, South Dakota, United States; University of Victoria, Victoria, British Columbia, Canada; Northern Arizona University, Northern Arizona University, Arizona, United States; Northern Arizona University, Flagstaff, Arizona, United States

## Abstract

Studying modifiable psychosocial risk factors for dementia is crucial to inform approaches for dementia prevention, especially among those with lower neighborhood quality. Perceived control is an important psychosocial resource for cognitive health. Most prior work, however, focuses exclusively on global aspects of control, ignoring influences of more dynamic aspects of control over specific experiences in daily life, such as daily stressor control. Therefore, we used data from the Midlife in the United States study and the National Study of Daily Experiences (N=1,651, Mage=62.09, SE=12.11, 56.65% Female) to address this gap and examine associations of stressor control and neighborhood quality with cognitive function. Over two waves of eight consecutive days of data collection, respondents reported perceived control over interpersonal and non-interpersonal stressors they had experienced. Respondents also completed assessments of neighborhood quality, executive function (EF), and episodic memory (EM). We employed linear regressions to assess concurrent (wave 2) and prospective (ten years later at wave 3) associations of stressor control and neighborhood quality with cognitive function. Concurrent associations between better neighborhood quality and better cognitive function (EF, EM) were amplified among those with lower interpersonal stressor control. No prospective associations with cognitive function ten years later emerged. Results indicate that better neighborhood quality may be beneficial for cognitive health, especially for individuals with lower perceived control over interpersonal stressors such as arguments and avoided arguments. We discuss the value of promoting better neighborhood quality as an important social determinant of cognitive health and aging when psychosocial resources are comparatively limited.

